# Serum albumin was negatively associated with diabetic peripheral neuropathy in Chinese population: a cross-sectional study

**DOI:** 10.1186/s13098-021-00718-4

**Published:** 2021-09-15

**Authors:** Pijun Yan, Qian Tang, Yuru Wu, Qin Wan, Zhihong Zhang, Yong Xu, Jianhua Zhu, Ying Miao

**Affiliations:** 1 Department of Endocrinology, The Affiliated Hospital of Southwest Medical University, Luzhou, 646000 Sichuan China; 2 Department of General Medicine, The Affiliated Hospital of Southwest Medical University, Luzhou, 646000 Sichuan China

**Keywords:** Albumin, Diabetic peripheral neuropathy, Type 2 diabetes mellitus, Vibration perception threshold

## Abstract

**Background:**

Previous studies that explored the relationship of serum albumin with diabetic peripheral neuropathy (DPN) have indicated inconsistent results. Thus, the present study aimed to evaluated the association between serum albumin and DPN, defined as vibration perception threshold (VPT) values ≥ 25 V and/or inability to feel the monofilament, in Chinese patients with type 2 diabetes mellitus (T2DM).

**Methods:**

1465 T2DM patients aged ≥ 16 years, who completed the measurement of serum albumin and DPN screening between 2012 and 2015, were included in the cross-sectional study. Correlation and multivariate logistic regression analysis models were used to evaluate the possible relationship between serum albumin and DPN.

**Results:**

Patients with higher quartiles of serum albumin had significantly lower VPT values and prevalence of DPN compared with those with lower quartiles (*P* for trend < 0.01), and there was an inverse relationship between serum albumin and VPT values and prevalence of DPN (all *P* < 0.01). Multivariate logistic regression analysis demonstrated that the risk of DPN was progressively decreased across serum albumin quartiles (*P* for trend < 0.01), and participants in the highest quartile of serum albumin were at a significantly decreased risk of DPN compared to those in the lowest quartile (odds rate: 0.311, 95% confidence intervals 0.134–0.724, *P* < 0.01). ROC analysis revealed that the optimal cutoff point of serum albumin for the prevalence of DPN was 39.95 g/L in patients with T2DM, with a sensitivity of 65.88% and a specificity of 66.7%.

**Conclusions:**

Decreased levels of serum albumin might be correlated with increased risk of DPN in Chinese patients with T2DM. Future longitudinal studies with large samples are warranted to confirm our findings, and elucidate putative mechanisms for the association.

**Supplementary Information:**

The online version contains supplementary material available at 10.1186/s13098-021-00718-4.

## Background

Diabetic peripheral neuropathy (DPN), characterized by numbness, sensory abnormalities and pain at an early stage, and sensory loss at advanced stages, has been recognized as one of the most frequent microvascular complications in diabetic subjects, affecting up to 50% such patients [1]. DPN is widely considered as a major risk factor for foot ulcers and even lower extremity amputation, which could result in considerable morbidity and mortality, and a high health care burden [2]. To date, its underlying pathogenetic mechanisms are unclear, and there are no effective treatment measures. Therefore, it is clinically important to early detect and treat novel modifiable risk factors that contribute to DPN.

Albumin is a 66.3 KD non-glycosylated protein, which is synthesized and secreted from liver cells, and considered to be a predominant protein component of blood plasma and extracellular compartment in humans. Many factors, including catabolic state, inflammation, synthesis rate, and distribution of intravascular and extravascular compartments, can influence serum albumin level [3, 4]. In recent years, it has been revealed that serum albumin plays an important role in the binding and transport many endogenous and exogenous substances including fatty acids, hormones, bilirubin, metal ions, foreign molecules such as drugs [5]. There is also extensive evidence that serum albumin has many physiological properties, including antioxidant, anti-inflammatory, antiplatelet agglutination, and anticoagulant activities, regulating immune response, preventing apoptosis of endothelial cell, Schwann cells and neuronal, dilating blood vessel, protecting neurons against ischemia and reperfusion-induced injury, and improving neuronal functional recovery [6–13]. Together, these findings suggest that albumin may confer robust neuroprotection, and lower levels of serum albumin may be involved in the development of DPN. Although some investigations have suggested that patients with DPN had significantly lower serum albumin [14, 15], and the associations of serum albumin with peripheral nerve function (composite Z scores of nerve conduction parameters) in the albuminuria group was independent of urinary microalbumin to creatinine ratio (ACR) [16], others have reported no association [17]. Such differences regarding the association between serum albumin and DPN in these literatures may be due to differences in study design and population, diabetic duration, diagnostic methods for DPN, confounding factors adjusted, and sample size.

Therefore, the present study aimed to examine the association between serum albumin and DPN in a Chinese population with T2DM. Moreover, we alsoevaluated the possible relationships among serum albumin and metabolic parameters, oxidative stress and inflammatory markers, and vascular related indicators.

## Methods

### Study population

The cross-sectional study finally enrolled 1465 confirmed or newly diagnosed T2DM patients in our inpatient department between August 2012 and September 2015, who completed the measurement of serum albumin and DPN screening. Inclusion criteria were long-term residence (≥ 5 years) in Sichuan province of China and age 16–89 years old. The exclusion criteria were as follows: (1) presence of other endocrine disorders, acute diabetic complications including diabetic ketoacidosis, hyperosmolar hyperglycemic nonketotic coma, and hypoglycemia, non-diabetic neuropathy; (2) any evidence of severe respiratory and cerebrovascular disease, severe congestive heart failure (New York Heart Association functional class IV), severe renal failure (serum creatinine ≥ 221 µmol/L), liver disease or abnormal liver function; (3) presence of inflammatory disease, autoimmune disease such as rheumatoid arthritis, ankylosing spondylitis, systemic lupus erythematosus, and synovitis, acute infectious disease, cancer, hematological diseases and cognitive dysfunction; (4) alcoholism, pregnancy or lactation; (5) use of immunosuppressant, antioxidant, anti-inflammatory drugs, analgesics, systemic corticosteroids; (6) use of possible or known drugs that may affect peripheral nerve function and sympathetic system; (7) Subjects with missing or incomplete data.

The study was performed in accordance with the ethical guidelines of the 1975 Declaration of Helsinki and was approved by the human research ethics committee of the Affiliated Hospital of Southwest Medical University, and informed consent was obtained from all patients after full explanation of the purpose and nature of our study.

### Clinical and biochemical measurements

Body weight and height were taken using standard protocols with the subjects in light clothing and without shoes. Body mass index (BMI) was calculated by dividing weight in kilograms by height in metres squared. Systolic and diastolic blood pressures were measured in all subjects on the right arm using a standard mercury sphygmomanometer [18]. All measurements were operated by trained study personnel.

In the morning, after an overnight fast of 8 h or longer, venous blood samples were collected from study participants to determine fasting blood glucose (FBG), glycated hemoglobin A1C (HbA1c), lipid profiles, including total cholesterol (TC), triglyceride (TG), high density lipoprotein cholesterol (HDL-C), and low-density lipoprotein cholesterol (LDL-C), alanine aminotransferase (ALT), aspartate aminotransferase (AST), gamma-glutamyltransferase (GGT), cystatin C (CysC), uric acid (UA), serum albumin, creatinine (Cr), neutrophil and lymphocyte counts, neutrophil to lymphocyte ratio (NLR), and red cell distribution width (RDW) at a certified central laboratory. FBG and HbA1c levels were measured by the glucose- oxidase method and anion exchange high performance liquid chromatography, respectively (arkray ELUENT 80A, Japan). Lipid profiles, ALT, AST, GGT, CysC, UA, serum albumin and Cr were analyzed using a 7060 full-automatic biochemical analyzer (Hitachi, Tokyo, Japan). Serum albumin level was determined using the bromocresol green method according to the manufacturer’s instruction, and its normal range was 40–55 g/L. The neutrophil and lymphocyte counts, NLR, and RDW were determined using an automated blood cell counter (Mindray BC-6800, Shenzhen, China). Estimated glomerular filtration rate (eGFR) was calculated according to serum Cr, gender, age, and race [19–22]. During the morning, spot urine samples were collected to measure microalbumin with immunoturbidimetric tests, and creatinine enzymatically. The urinary microalbumin to creatinine ratio (ACR; mg/g creatinine) was calculated by dividing urine microalbumin by urine creatinine.

### Measurements of ankle-brachial index (ABI) and diagnosis of DPN

Ankle-brachial index (ABI) values were measured by a continuous-wave Doppler ultrasound probe (Vista AVS, Summit Co.) and vibration perception thresholds (VPT) value was performed by a neurothesiometer (Bio-Thesiometer; Bio-Medical Instrument Co., Newbury, OH, USA) as previously described [19, 20]. Touch sensation was assessed by a 10 g Semmes–Weinstein monofilament. All measurements were operated by the same experienced physician. Well-trained professionals used a standard questionnaire and face-to-face interviews to obtain information on participants’ neuropathy symptoms, including feelings of burning, numbness, tingling, pain, fatigue, and paresthesia, and performed the clinical examinations. DPN was defined as VPT values of 25 V or more and/or inability to feel the monofilament [19].

### Statistical analysis

All statistical analyses were performed using the SPSS 20.0 software (Chicago, IL). All data were first analyzed for normality of distribution using the Kolmogorov–Smirnov test of normality, and homogeneity of variance. Normally distributed continuous variables are presented as mean ± standard deviation (SD) and nonparametric distributed continuous variables are presented as median (25th percentile–75th percentile). Categorical variables are express as numbers (percentages).

Serum albumin quartiles were categorized as follows: Q1 (21.70–37.60 g/L), Q2 (37.70–41.30 g/L), Q3 (41.40–44.40 g/L), and Q4 (44.50–57.60 g/L).

Moreover, all participants were divided into DPN group (n = 231) and no DPN group (n = 1234). A previous work has proposed that the mean ± SD of serum albumin in T2DM patients with and without DPN is 42.51 ± 2.88 g/L and 44.28 ± 3.56 g/L, respectively [16]. We set type I error α at 0.05 and type II error β at 0.1, and assume that the sample size ratio between the T2DM patients with DPN group and the T2DM patients without DPN group is 1: 5.5. Based on these assumptions and a previous work, sample size calculations indicated that a total of 269 patients (41 T2DM patients with DPN, and 228 T2DM patients without DPN) were required for the study. In other words, this study would provide 90% statistical power as long as the sample size in the T2DM patients without DPN group is ≥ 228 and the sample size in the T2DM patients with DPN group is ≥ 41.

Differences in clinical and biochemical parameters between two groups or among three or more groups were compared by Student’s t-test, Mann–Whitney U test, One-way analysis of variance (ANOVA), Kruskal–Wallis test, or *χ*^*2*^ test. Associations between serum albumin and other variables were tested by the Spearman’s correlation analysis and also partial correlation analyses controlling for gender, age, BMI and diabetic duration. A binary logistic regression analysis was conducted to estimate the odds ratios (ORs) for DPN according to quartiles of serum albumin in type 2 diabetes. Model 1 was unadjusted. Model 2 was adjusted for age, gender, and BMI, and diabetic duration. Model 3 was additionally adjusted for SBP, DBP, FBG, HbA1c, TC, TG, HDL-C, LDL-C, NLR, RDW, ALT, AST, GGT, UA, serum Cr, CysC, eGFR, ACR, and ABI. The Q1 served as the reference group, and Odds ratios (OR) and 95% confidence intervals (CI) were estimated. Receiver operating characteristic (ROC) curve analysis was performed to identify the optimal cutoff values for serum albumin as indicators of DPN.

In all statistical tests, a two-sided *P* < 0.05 was considered statistically significant.

## Results

### Clinical and biochemical characteristics of the study population

The basic characteristics of the study population are summarized in Table 1. Among the included participants (n = 1465), 15.77% (n = 231) had DPN. The prevalence of DPN according to serum albumin quartiles was 31.25, 16.76, 9.81 and 5.19%, respectively. The diabetic subjects with higher serum albumin were more likely to be younger, and had longer diabetic duration (all *P* < 0.01). The diabetic subjects in the higher serum albumin quartiles exhibited higher levels of BMI, DBP, TG, TC, HDL-C, LDL-C, ALT, AST, UA, eGFR, ABI, and lower levels of FBG, HbA1c, GGT, serum Cr, CysC, ACR, NLR, VPT values and prevalence of DPN compared with those in the lowest quartile (*P* < 0.01 or *P* < 0.05). When compared with those without DPN, subjects with DPN had significantly older age, longer diabetic duration, higher levels of FBG, HbA1c, CysC, serum Cr, urinary ACR, NLR, VPT values, and lower levels of serum albumin, BMI, DBP, TC, TG, ALT, AST, GGT, eGFR and ABI (*P* < 0.01 or *P* < 0.05) (Additional file 1: Table S1).

**Table 1:** Clinical and biochemical characteristics of study participants according to serum albumin quartiles

Characteristics	Q1	Q2	Q3	Q4	*P*
	21.70–37.60	37.70–41.30	41.40–44.40	44.50–57.60	
	(n = 368)	(n = 364)	(n = 367)	(n = 366)	
Albumin (g/L)	35.50 (33.10–36.80)	39.70 (38.80–40.50)	42.70 (42.00–43.50)	46.60 (45.40–48.03)	0.000
Male/Female	185/183	177/187	179/188	183/183	0.958
Age (years)	62.75 ± 11.13	60.63 ± 11.25	58.89 ± 11.23	57.36 ± 11.02	0.000
BMI (kg/m^2^)	23.05 ± 3.40	24.57 ± 4.21	24.59 ± 3.60	24.73 ± 3.48	0.000
Diabetic duration (years)	8.00 (3.00–12.00)	8.00 (3.00–11.00)	6.00 (2.00–10.00)	6.00 (2.00–10.00)	0.002
SBP (mmHg)	131.50 (118.00–149.00)	130.00 (116.25–144.75)	130.00 (119.00–145.00)	129.00 (119.00–140.25)	0.434
DBP (mmHg)	70.58 ± 11.73	71.54 ± 12.46	71.99 ± 11.81	73.84 ± 12.40	0.004
FBG (mmol/L)	10.32 (7.41–14.59)	9.14 (6.80–13.16)	9.65 (6.90–13.17)	8.94 (7.09–12.19)	0.022
HbA1c (%)	10.20 (8.10–12.20)	9.30 (7.80–11.10)	8.90 (7.30–10.80)	8.30 (7.10–10.10)	0.000
TC (mmol/L)	4.26 (3.50–5.23)	4.54 (3.83–5.35)	4.86 (4.16–5.63)	4.98 (4.30–5.72)	0.000
TG (mmol/L)	1.32 (0.94–1.98)	1.66 (1.18–2.46)	1.81 (1.21–2.77)	1.93 (1.30–3.09)	0.000
HDL-C(mmol/L)	1.09 (0.89–1.31)	1.08 (0.91–1.27)	1.16 (0.96–1.36)	1.20 (1.00–1.42)	0.000
LDL-C(mmol/L)	2.45 (1.87–3.23)	2.56 (2.09–3.24)	2.76 (2.18–3.34)	2.87 (2.19–3.47)	0.000
ALT (U/L)	14.10 (10.13–22.00)	16.60 (12.00–23.80)	19.90 (14.00–29.00)	20.90 (15.68–32.00)	0.000
AST (U/L)	16.35 (12.70–21.38)	17.05 (14.00–21.33)	19.20 (15.40–24.90)	21.10 (17.28–27.30)	0.000
GGT (U/L)	20.15 (13.60–36.80)	23.05 (15.53–39.50)	26.80 (16.80–42.40)	28.45 (19.30–48.03)	0.000
UA (μmol/L)	290.70 (221.05–358.33)	297.70 (243.12–369.12)	308.15 (247.18–373.58)	319.60 (267.90–393.70)	0.000
CysC (mg/L)	1.01 (0.76–1.43)	0.88 (0.71–1.16)	0.86 (0.71–1.05)	0.82 (0.71–0.99)	0.000
Serum Cr (μmol/L)	68.20 (53.93–97.95)	61.90 (51.10–81.28)	63.10 (50.80–76.20)	61.35 (50.68–73.73)	0.000
eGFR (mL/min/1.73 m^2^)	88.78 (60.95–103.96)	97.62 (77.30–107.54)	97.43 (82.73–109.14)	100.14 (88.51–110.31)	0.000
ACR (mg/g)	60.50 (19.60–481.70)	25.43 (13.15–68.14)	23.10 (11.21–56.24)	19.17 (8.78–42.78)	0.000
NLR	3.36 (2.11–4.71)	2.58 (1.90–3.66)	2.43 (1.79–3.48)	2.41 (1.74–3.31)	0.000
RDW	13.05 (12.50–13.70)	12.90 (12.40–13.60)	12.90 (12.50–13.60)	13.00 (12.50–13.63)	0.501
ABI	1.04 (0.97–1.11)	1.04 (1.00–1.09)	1.05 (1.00–1.11)	1.05 (1.00–1.10)	0.033
VPT (V)	16.00 (12.00–28.00)	14.00 (10.00–20.00)	12.00 (10.00–18.00)	12.00 (8.00–16.00)	0.000
DPN (n, %)	115 (31.25)	61 (16.76)	36 (9.81)	19 (5.19)	0.000

Data are mean ± standard deviation for continuous variables or n (percentage) for categorical variables. *BMI* body mass index, *SBP* systolic blood pressure, *DBP* diastolic blood pressure, *FBG* fasting blood glucose, *HbA1c* glycated hemoglobin A1c, *TC* total cholesterol, *TG* triglyceride, *HDL-C* high-density lipoprotein cholesterol, *LDL-C* low-density lipoprotein cholesterol, *NLR* neutrophil to lymphocyte ratio, *ALT* alanine aminotransferase, *AST* aspartate aminotransferase, *GGT* gamma-glutamyltransferase, *UA* uric acid, *CysC* cystatin C, *Cr* creatinine, *eGFR* estimated glomerular filtration rate; ACR, albumin- to-creatinine ratio, *NLR* neutrophil to lymphocyte ratio, *RDW* red cell distribution width, *ABI* Ankle-brachial index, *VPT* vibration perception threshold; *DPN* diabetic peripheral neuropathy

### Association between serum albumin and risk factors related to DPN in study subjects

The Spearman correlation analysis revealed that serum albumin levels were positively associated with BMI, DBP, TC, TG, HDL-C, LDL-C, ALT, AST, GGT, UA, eGFR, ABI, and negatively with age, diabetic duration, FBG, HbA1c, serum Cr, CysC, ACR, NLR, VPT values and prevalence of DPN (*P* < 0.01 or *P* < 0.05; Table 2). After adjusting for age, gender, BMI and diabetic duration, the associations among serum albumin and metabolic parameters (DBP, TC, TG, HDL-C, HbA1c), inflammation and oxidative stress parameters (NLR, UA and GGT), and diabetic nephropathy (DN) related parameters (serum Cr, CysC and ACR) remained statistically significant (*P* < 0.01 or *P* < 0.05; Table 2).

**Table 2:** Association between serum albumin and risk factors related to DPN in study subjects

Variable	r	*P*-value	Adjusted r	Adjusted *P*-value
Age	− 0.165	0.000	–	–
Gender	− 0.009	0.742	–	–
BMI	0.179	0.000	–	–
Diabetic duration	− 0.086	0.001	–	–
SBP	− 0.036	0.170	− 0.025	0.563
DBP	0.101	0.000	0.107	0.013
FBG	− 0.076	0.004	− 0.042	0.336
HbA1c	− 0.238	0.000	− 0.217	0.000
TC	0.216	0.000	0.167	0.000
TG	0.230	0.000	0.223	0.000
HDL-C	0.129	0.000	0.203	0.000
LDL-C	0.127	0.000	0.077	0.077
ALT	0.283	0.000	0.006	0.898
AST	0.261	0.000	− 0.045	0.298
GGT	0.148	0.000	− 0.091	0.036
UA	0.139	0.000	0.159	0.000
CysC	− 0.184	0.000	− 0.123	0.004
Serum Cr	− 0.126	0.000	− 0.120	0.005
eGFR	0.183	0.000	0.084	0.053
ACR	− 0.284	0.000	− 0.338	0.000
NLR	− 0.196	0.000	− 0.154	0.000
RDW	− 0.001	0.963	0.020	0.647
ABI	0.066	0.012	0.009	0.829
VPT	− 0.313	0.000	− 0.314	0.000
DPN	− 0.278	0.000	− 0.241	0.000

### Association between serum albumin quartiles and the risk of DPN in study subjects

The mean serum albumin in study population was 40.93 g/L (range 21.70–57.60 g/L). There was a 53.1% decreased risk of prevalent DPN per SD increase in serum albumin (OR = 0.469, 95% CI 0.404–0.544) in Model 1. Results remained significant after further adjusting for demographic, metabolic, inflammation and oxidative stress, and other parameters in Model 3 (OR = 0.499, 95% CI 0.385–0.645, *P* < 0.01). Moreover, the risk of DPN was progressively decreased with increasing serum albumin quartiles in Model 1–3 (*P* for trend < 0.01), and participants in the highest quartile of serum albumin were at a significantly decreased risk of DPN compared to those in the lowest quartile (odds rate: 0.311, 95% confidence intervals 0.134–0.724, *P* < 0.01) (Table 3).

**Table 3:** Association between serum albumin quartiles and the risk of DPN in study subjects

		DPN	
Serum albumin (g/L)	Model 1	Model 2	Model 3
	OR (95%CI)	OR (95%CI)	OR (95%CI)
Per SD increase	0.469 (0.404–0.544)	0.505 (0.429–0.595)	0.499 (0.385–0.645)
Quartiles of albumin			
Q1 (21.70–37.60)	1	1	1
Q2 (37.70–41.30)	0.443 (0.311–0.630)	0.485 (0.329–0.714)	0.683 (0.416–1.122)
Q3 (41.40–44.40)	0.239 (0.159–0.360)	0.298 (0.192–0.463)	0.475 (0.251–0.900)
Q4 (44.50–57.60)	0.120 (0.072–0.201)	0.174 (0.102–0.297)	0.311 (0.134–0.724)
*P* for trend	0.000	0.000	0.000
Q4 versus Q1, Q2, Q3	0.000	0.000	0.003

Model 1: unadjusted; Model 2: adjusted for age, gender, BMI, and diabetic duration; Model 3: adjusted for age, sex, BMI, diabetic duration, SBP, DBP, FBG, HbA1c, TC, TG, HDL-C, LDL-C, NLR, RDW, ALT, AST, GGT, UA, serum Cr, CysC, eGFR, ACR and ABI

### Predictive value of serum albumin for the prevalence of DPN in patients with T2DM

**Fig.1:**
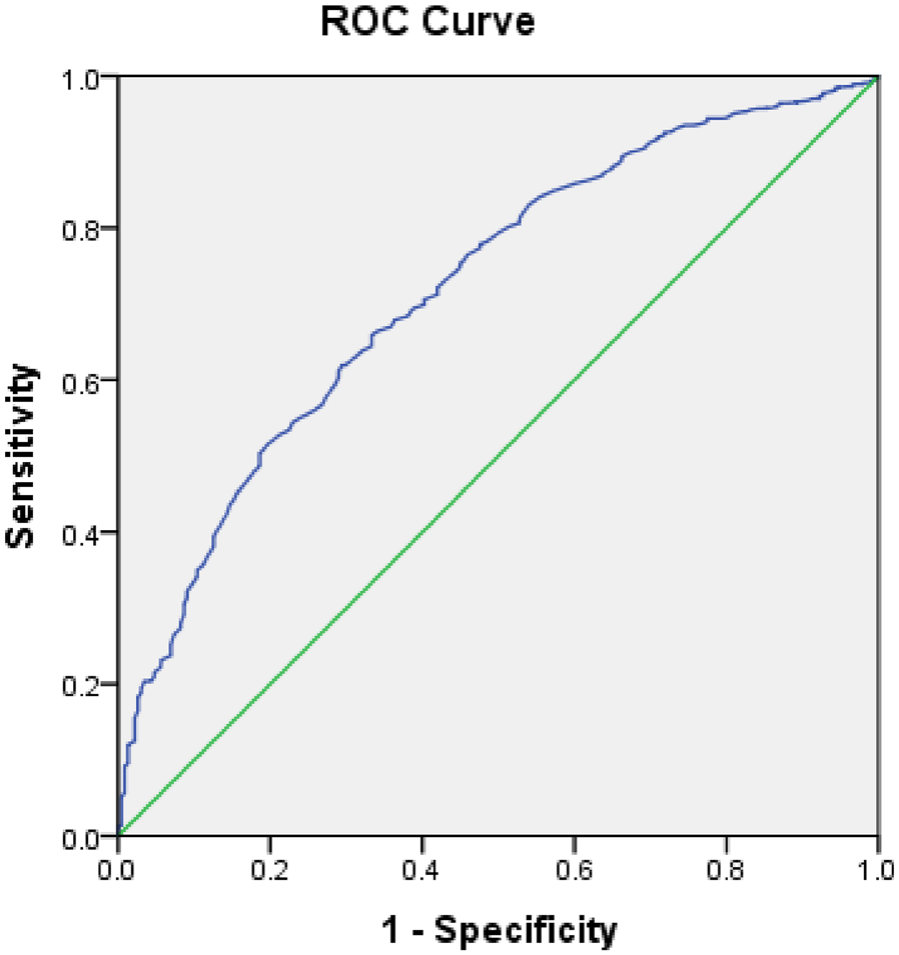
ROC analysis of serum albumin to indicate DPN for T2DM individuals. AUC = 0.720; 95% CI, 0.685–0.755; *P* < 0.001; identified serum albumin cutoff value = 39.95 g/L; Youden index = 0.326; sensitivity: 65.88%; specificity: 66.7%

ROC analysis revealed that the optimal cutoff point of serum albumin was 39.95 g/L for the prevalence of DPN (AUC = 0.720; 95% CI, 0.685–0.755; Youden index = 0.326; sensitivity, 65.88%; specificity, 66.7%) in diabetic patients (Fig. 1).

## Discussion

In the present study, we found that patients in the highest quartile of serum albumin had lower prevalence of DPN compared with those in the lowest quartile (*P* < 0.01), and serum albumin showed a negative association with VPT values and prevalence of DPN (all *P* < 0.01). After multivariate adjustment, the risk of DPN was progressively decreased with increasing serum albumin quartiles (*P* for trend < 0.01), and participants in the highest quartile of serum albumin were at a significantly decreased risk of DPN compared to those in the lowest quartile (OR: 0.311, 95% CI 0.134–0.724, *P* < 0.01). ROC analysis revealed that the optimal cutoff point of serum albumin for the prevalence of DPN was 39.95 g/L in patients with T2DM. These findings suggest that lower serum albumin level might be at increased risk of DPN.

Albumin is a multifunctional protein in plasma that binds metal ions, effectively scavenges free oxygen and nitrogen radicals, subsequently resulting in important antioxidant properties [5]. It has been reported that serum albumin, a chain-breaking antioxidant in vitro, has stronger antioxidant capacity compared to bilirubin, vitamin C and E [23]. Changes of circulating concentration and structural modification of albumin induced by hyperglycemia or free radicals damage its antioxidant effects, which eventually lead to increased oxidative stress [24]. Several previous studies have explored the association of serum albumin with DPN, and provided inconsistent results [14–17]. In a study of 130 Japanese patients with T2DM conducted by Iwasaki et al., serum albumin was found to be independently inversely associated with the presence and severity of DPN, as evidence by decreased median motor nerve conduction velocity and increased minimum F-wave latency of median and tibial nerve [16]. Data from a cross-sectional study of 409 Chinese T2DM patients underwent measurement of nerve conduction demonstrated that serum albumin showed an independently positively association with composite Z scores of conduction velocity and amplitude, and negatively with composite Z scores of latency in T2DM patients, especially in those with albuminuria [14]. Recently reported data from the Saudi study, which included 2906 hospitalized adult diabetics, demonstrated that patients with DPN had significantly lower serum albumin compared with those without [15]. However, no statistically significant association between serum albumin and DPN was found in US diabetic patients undergoing foot and ankle surgery over a 13 month period [17]. In the present study, we observed that patients in higher quartile of serum albumin had lower VPT values, an useful and reliable method for early screening DPN and reflecting the clinical severity [19], and prevalence of DPN, and serum albumin showed a negatively association with VPT values and prevalence of DPN, demonstrating the potential neuroprotective effects of serum albumin on the development of DPN possibly due to its antioxidant, anti-inflammatory, and anti-atherosclerosis. Moreover, after multivariate adjustment, the risk of DPN per SD increase in serum albumin was reduced by 50.1%, and the risk of DPN was progressively decreased with increasing serum albumin quartiles. Further ROC analysis revealed that the optimal cutoff point of serum albumin for the prevalence of DPN was 39.95 g/L in patients with T2DM. Collectively, these data demonstrated that low serum albumin might be associated with DPN possibly due to deceased neuroprotective effects in Chinese patients with T2DM.

There is mounting evidence suggesting that low-grade inflammation and oxidative stress induced by chronic hyperglycemia have potential roles in the pathogenesis and progression of DPN [14, 25]. Our study provided further evidence that patients with DPN had severely impaired glycemic control (higher FBG and HbA1c) and dysregulation of inflammation and oxidative stress balance (higher CysC, NLR, and lower GGT) [19, 26–28] compared to those without, mostly consistent with those findings of previous studies [19, 25, 29], supporting a potential role of hyperglycemia-induced inflammation and oxidative stress in the development of DPN. Moreover, we revealed that T2DM subjects in the highest quartiles of serum albumin exhibited better glycemic control (lower FBG and HbA1c), and lower inflammation and oxidative stress balance (decrease CysC, NLR, GGT, and increased UA [20]), and serum albumin levels were positively associated with UA and negatively with HbA1c, GGT, NLR and CysC, indicating that as a negative acute phase protein, serum albumin may possess anti-inflammatory and antioxidant effects, and low serum albumin might be associated with DPN. Yeh et al. performed a retrospective study of 252 adult patients receiving enteral nutrition in a surgical intensive care unit, and found that serum albumin had a negative association with proinflammatory cytokines, such as C-reactive protein (CRP), white blood cell, and especially NLR [30]. Experiments showed that albumin could protect cells against oxidant injury and control inflammatory through specifically regulating levels of cellular glutathione (GSH) and decreasing TNF-α-induced activation of NF-kappaB [31]. In human aortic endothelial cells, Zhang et al. also reported that physiological albumin levels selectively inhibited the expression of vascular cell adhesion molecule-1 induced by TNF-ɑ in a GSH-independent manner [32]. These findings of previous and our studies suggest that serum albumin may exert antioxidant and anti-inflammatory effects, and increased inflammation and oxidative stress due to low serum albumin may be the main mechanism underlying the association of serum albumin with DPN, and further studies are needed to confirm our speculation.

A growing body of literature suggests that diabetic vasculopathy due to low-grade inflammation and oxidative stress, which could affect the nutrition and blood supply of neuronal and Schwann cells and subsequently contribute to nerve degeneration, is believed to play an important role in the pathogenesis of DPN [19]. DN, a diabetic microvascular complication characterized by a decreased eGFR, increased urinary ACR and CysC, and high levels of serum Cr [33], is independently associated with DPN [26, 34–36]. ABI is a quick and easy way to detect whether the patient has peripheral artery disease (PAD), and a useful marker for atherosclerosis at other vascular sites [20]. Our study provided further evidence that supported the potential role of diabetic vasculopathy, especially DN and PAD, in the development of DPN, since we found that patients with DPN had higher CysC, serum Cr, urinary ACR, and lower eGFR and ABI. Moreover, we observed that T2DM subjects in the highest serum albumin quartiles exhibited higher levels of eGFR, ABI, and lower serum Cr, CysC and ACR compared with those in the lowest quartile, and serum albumin was negatively associated with serum Cr, CysC, and ACR, indicating that serum albumin levels may be associated with DN and PAD as diabetic micro- and macrovascular complications, respectively, which were consistent with the results of previous reports [17, 37–39]. A follow-up study of 188 patients with T2DM and biopsy-proven DN conducted by Zhang et al. showed significantly associations among serum albumin and glomerular lesions, proteinuria, renal function, and patients with hypoalbuminemia had a poorer renal prognosis [37]. Decreased concentration of serum albumin in patients with DN was confirmed by another cross-sectional study in japan [38]. Recently, Greenhagen and collaborators showed lower serum albumin in US subjects with PAD [17]. More recently, data from another cross-sectional study in China of 10,900 hypertensive patients aged ≥ 18 years demonstrated that in men, serum albumin was significantly inversely related to the prevalence of PAD [39]. Together, our results and previous findings implied that a negative association between serum albumin and DPN appeared to be due to the systemic circulation damage, especially DN and PAD, subsequently leading to adequate nutrition and blood supply of neurons and nerves in such individuals.

Our study has several possible limitations. First, the cross-sectional design of our study can't infer causality. Thus, prospective investigation is needed to confirm our findings. Second, we only used UA, GGT, CysC and NLR to evaluate inflammation and oxidative stress because data about classical inflammatory and oxidative stress markers (TNF-α, CRP and GSH**)** is lacking in our study, which makes it difficult to draw definite and consistent conclusion. Third, although many confounding factors were adjusted, it is possible that there were still several residual confounding and unmeasured factors that may affect the true association of serum albumin with DPN. Fourth, ROC analysis revealed that a serum albumin level of ≥ 39.95 g/L could predict the prevalence of DPN, but the power of discrimination found relatively low (sensitivity of 65.88% and a specificity of 66.7%), which may be related to small sample size in DPN group and suspected cases between the two groups. Therefore, in later work, we would improve the diagnostic value of serum albumin by increasing sample size and improve diagnostic methods of DPN. Fifth, broad exclusion criteria and sample from a single center may limit the external validity of the study. In spite of some limitations, our study has some strength, including a relatively large sample size, a thorough clinical and laboratory assessment and appropriate adjustment.

## Conclusions

Our data suggests that in Chinese patients with type 2 diabetes, low serum albumin may be independently related to the risk of DPN, which may be due to the reduced anti-inflammatory, anti-oxidant and anti-atherosclerotic effects. Further, well-designed larger-scale prospective cohort studies are needed to provide more convincing conclusions.

## Supplementary Information

**Additional file 1: Table S1.** Characteristics of the 1465 patients by DPN.

## Data Availability

The data is available upon reasonable request to the corresponding author.

## Abbreviations

DPN: Diabetic peripheral neuropathy
T2DM: Type 2 diabetes mellitus
BMI: Body mass index
SBP: Systolic blood pressure
DBP: Diastolic blood pressure
FBG: Fasting blood glucose
HbA1c: Glycated hemoglobin A1c
TC: Total cholesterol
TG: Triglyceride
HDL-C: High-density lipoprotein cholesterol
LDL-C: Low-density lipoprotein cholesterol
ALT: Alanine aminotransferase
AST: Aspartate aminotransferase
GGT: Gamma-glutamyltransferase
UA: Uric acid
CysC: Cystatin C
Cr: Creatinine
NLR: Neutrophil to lymphocyte ratio
RDW: Red cell distribution width
eGFR: Estimated glomerular filtration rate
ACR: Albumin- to-creatinine ratio
ABI: Ankle-brachial index
VPT: Vibration perception threshold
SD: Standard deviation
ANOVA: One-way analysis of variance
Q: Quartile
CI: Confidence intervals
ROC: Receiver operating characteristic
CRP: C-reactive protein
TNF-α: Tumor necrosis factor-α
GSH: Glutathione
DN: Diabetic nephropathy
PAD: Peripheral arterial disease
