# Supplementary material for: Serum albumin was negatively associated with diabetic peripheral neuropathy in Chinese population: a cross-sectional study
Source: Diabetol Metab Syndr. 2021 Sep 15;13:100. doi: 10.1186/s13098-021-00718-4 (PMC8444578; doi:10.1186/s13098-021-00718-4)
Supplement: Supplementary file 1 — Additional file 1: Table S1. Characteristics of the 1465 patients by DPN. [file 13098_2021_718_MOESM1_ESM.doc]

**Additional file 1: Table S1 Characteristics of the 1465 patients by DPN.**

| **Variables** | **no DPN** | **DPN** | ***P*** |
| --- | --- | --- | --- |
|  | **(n =1234 )** | **(n = 231)** |  |
| Male/Female | 598/636 | 126/105 | 0.090 |
| Age (years) | 59.00 (50.00–67.00) | 66.00 (61.00–74.00) | 0.000 |
| BMI (kg/m^2^) | 24.09 (21.87–26.57) | 23.41 (21.16–25.66) | 0.002 |
| Diabetic duration (years) | 6.00 (2.00–10.00) | 10.00 (5.00–15.00) | 0.000 |
| SBP (mmHg) | 130.00 (118.00–144.00) | 132.00 (119.00–151.00) | 0.068 |
| DBP (mmHg) | 72.00 (64.75–80.00) | 68.00 (62.00–78.00) | 0.002 |
| FBG (mmol/L) | 9.20 (6.93–12.99) | 10.77 (7.70–14.43) | 0.003 |
| HbA1c (%) | 9.10 (7.40–11.00) | 9.80 (8.20–11.70) | 0.000 |
| TC (mmol/L) | 4.72 (4.00–5.53) | 4.47 (3.68–5.33) | 0.004 |
| TG (mmol/L) | 1.68 (1.15–2.67) | 1.49 (1.01–2.17) | 0.001 |
| HDL-C (mmol/L) | 1.13 (0.94–1.35) | 1.13 (0.91–1.34) | 0.536 |
| LDL-C (mmol/L) | 2.67 (2.08–3.34) | 2.59 (2.02–3.31) | 0.445 |
| ALT (U/L) | 18.40 (13.10–28.60) | 14.10 (10.30–21.30) | 0.000 |
| AST (U/L) | 18.50 (15.00–24.50) | 16.60 (13.40–21.90) | 0.000 |
| GGT (U/L) | 24.75 (16.48–42.30) | 23.00 (14.30–38.90) | 0.027 |
| Serum albumin (g/L) | 41.53±4.77 | 37.75±4.73 | 0.000 |
| UA (μmol/L) | 303.20 (245.50–368.90) | 317.95 (248.60–390.97) | 0.073 |
| CysC (mg/L) | 0.84 (0.70–1.06) | 1.09 (0.85–1.60) | 0.000 |
| Serum Cr (μmol/L) | 61.90 (50.58–77.00) | 73.60 (56.30–105.70) | 0.000 |
| eGFR (mL/min/1.73 m^2^) | 98.48 (83.37–109.63) | 83.72 (55.01–99.67) | 0.000 |
| ACR (mg/g) | 23.47 (11.14–60.33) | 66.30 (20.80–319.25) | 0.000 |
| NLR | 2.48 (1.79–3.55) | 3.59 (2.47–5.02) | 0.000 |
| RDW | 13.00 (12.50–13.60) | 13.00 (12.50–13.80) | 0.228 |
| VPT (V) | 12.00 (10.00–16.00) | 34.00 (28.00–46.00) | 0.000 |
| ABI | 1.05 (1.00–1.10) | 1.01 (0.85–1.08) | 0.000 |

Data are mean ± SD. SD, standard deviation; BMI, body mass index; SBP, systolic blood pressure; DBP, diastolic blood pressure; FBG, fasting blood glucose; HbA1c, glycated hemoglobin A1c; TC, total cholesterol; TG, triglyceride; HDL-C, high-density lipoprotein cholesterol; LDL-C, low-density lipoprotein cholesterol;

ALT, alanine aminotransferase; AST, aspartate aminotransferase; GGT, gamma-glutamyltransferase; UA, uric acid; CysC, cystatin C; Cr, creatinine; eGFR, estimated glomerular filtration rate; ACR, albumin- to-creatinine ratio; NLR, neutrophil to lymphocyte ratio; RDW, red cell distribution width; VPT, vibration perception threshold; ABI, Ankle-brachial index.
